# *N*-(2-Bromo­phen­yl)-4-methyl-*N*-(4-methyl­phen­ylsulfon­yl)benzene­sulfonamide

**DOI:** 10.1107/S1600536811032533

**Published:** 2011-08-17

**Authors:** Muhammad Nadeem Arshad, Islam Ullah Khan, K. Travis Holman, Abdullah M. Asiri, H. M. Rafique

**Affiliations:** a X-ray Diffraction and Crystallography Laboratory, Department of Physics, School of Physical Sciences, University of the Punjab, Quaid-e-Azam Campus, Lahore-54590, Pakistan; b Materials Chemistry Laboratory, Department of Chemistry, GC University, Lahore-54000, Pakistan; c Department of Chemistry, Goergetown University, 37th and Oth Street NW, Washington, DC 20057, USA; d The Center of Excellence for Advanced Materials Research, King Abdul Aziz University, Jeddah, PO Box 80203, Saudi Arabia

## Abstract

In the title compound, C_20_H_18_BrNO_4_S_2_, the mean planes formed by the toluene substituents are inclined at a dihedral angle of 45.34 (8)°. The bromo­benzene group is disordered over two positions with an occupancy ratio of 0.74:0.26, resulting in two conformations of the ring; the two rings are oriented at a dihedral angle of 6.6 (6)° with each other. In the crystal structure, weak C—H⋯O inter­actions connect the mol­ecules in a zigzag manner along the *a* axis.

## Related literature

For general background, see: Ames & Opalko (1984 ▶); Arshad *et al.* (2011 ▶). For related structures, see: Zhao *et al.* (2007 ▶); Song (2008 ▶); Hanson & Hitchcock (2004 ▶).
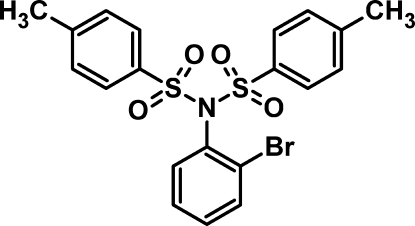


## Experimental

### 

#### Crystal data

C_20_H_18_BrNO_4_S_2_
*M* *_r_* = 480.38
Monoclinic, 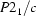

*a* = 10.5819 (15) Å
*b* = 13.1465 (19) Å
*c* = 14.235 (2) Å
β = 95.478 (2)°
*V* = 1971.2 (5) Å^3^
*Z* = 4
Mo *K*α radiation
μ = 2.32 mm^−1^
*T* = 100 K
0.38 × 0.33 × 0.24 mm

#### Data collection

Bruker KAPPA APEXII CCD diffractometer
Absorption correction: multi-scan (*SADABS*; Bruker, 2001 ▶) *T*_min_ = 0.472, *T*_max_ = 0.605
23193 measured reflections
4792 independent reflections
4320 reflections with *I* > 2σ(*I*)
*R*_int_ = 0.031

#### Refinement

*R*[*F* ^2^ > 2σ(*F* ^2^)] = 0.038
*wR*(*F* ^2^) = 0.085
*S* = 1.24
4792 reflections
301 parameters
H-atom parameters constrained
Δρ_max_ = 0.52 e Å^−3^
Δρ_min_ = −0.53 e Å^−3^

### 

Data collection: *APEX2* (Bruker, 2001 ▶); cell refinement: *SAINT* (Bruker, 2001 ▶); data reduction: *SAINT*; program(s) used to solve structure: *SHELXS97* (Sheldrick, 2008 ▶); program(s) used to refine structure: *SHELXL97* (Sheldrick, 2008 ▶); molecular graphics: *PLATON* (Spek, 2009 ▶) and *X-SEED* (Barbour, 2001 ▶); software used to prepare material for publication: *WinGX* (Farrugia, 1999 ▶).

## Supplementary Material

Crystal structure: contains datablock(s) I, global. DOI: 10.1107/S1600536811032533/pv2443sup1.cif

Structure factors: contains datablock(s) I. DOI: 10.1107/S1600536811032533/pv2443Isup2.hkl

Supplementary material file. DOI: 10.1107/S1600536811032533/pv2443Isup3.cml

Additional supplementary materials:  crystallographic information; 3D view; checkCIF report

## Figures and Tables

**Table 1:** Hydrogen-bond geometry (Å, °)

*D*—H⋯*A*	*D*—H	H⋯*A*	*D*⋯*A*	*D*—H⋯*A*
C5—H5⋯O3^i^	0.95	2.45	3.199 (3)	135

Symmetry code: (i) 
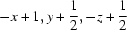
.

**Fig. 1.**
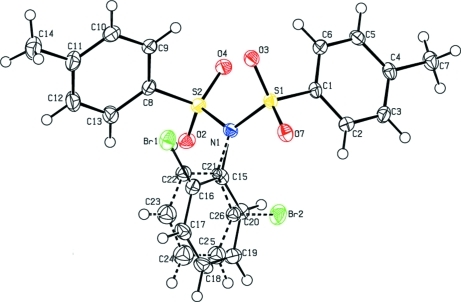
An ORTEP diagram of the title compound with thermal ellipsoids drawn at the 50% probability level; smaller fraction of the disordered bromobenzene ring has been plotted with dashed lines.

**Fig. 2.**
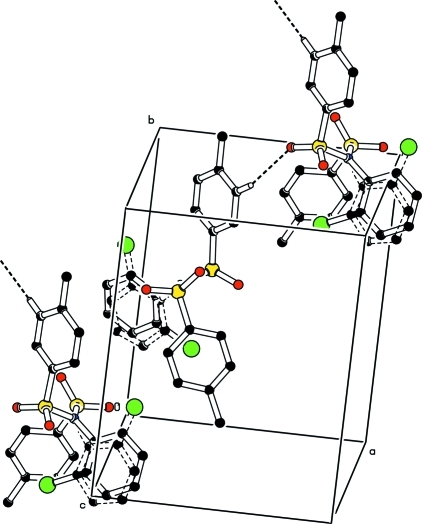
Unit cell packing for the title compound showing weak C—H···O interactions as dashed lines.

## supplementary crystallographic information

### Comment

*O*-Bromoaryl sulfonamides have been used for intramolecular arylation via palladium catalysis (Ames & Opalko, 1984). Herein, we report the crystal structure of the title compound which was synthesised as a precursor of cyclic sultams (Arshad *et al.*, 2011).

The *N*-atom of *O*-bromoaniline is directly attached to two p-toluene sulfonyl moieties. The interesting feature in the crystal structure is that bromobenzene group is disordered over two positions (C15—C20/Br1) and (C21—C26/Br2) with the occupancy of 0.74 and 0.26, respectively. The dihedral angle between the two disordered parts of the ring is 6.6 (6)°. The two toluene rings (C1—C7) & (C8—C14) are oriented at dihedral angle of 45.34 (89)°. The part (C15—C20/Br1) of bromobenzene ring formed dihedral angles of 24.0 (2)° and 38.77 (11)° with both of the toluene rings (C1—C7) & (C8—C14,) respectively, while the other part is oriented at dihedral angles of 19.6 (6)° and 34.8 (2)° with respect to the toluene rings. No classical hydrogen bonding has been observed in the molecule, only C—H···O type interactions connect the molecules in a zig-zag mode (Fig. 2 and Tab. 1).

### Experimental

A mixture of 2-bromoaniline (300 mg, 1.7 mmol) and triethylamine (529 mg, 5.2 mmol) was prepared in dichloromethane (20ml). Toluene sulfonylchloride (650 mg, 3.4 mmol) was added to the mixture and stirred for about two hours. The mixture was poured on ice and pH was adjusted about 2-3. The precipitate obtained was filtered, washed and dried. Suitable crystals were produced in methanol by slow evaporation.

### Refinement

The H-atoms were positioned at idealized geometry with C—H = 0.95 and 0.98 Å for aryl and methyl groups, respectively, and were refined using a riding model with *U*_iso_(H) = 1.2 *U*_eq_(C) for aromatic & *U*_iso_(H) = 1.5 *U*_eq_(C) for methyl groups. The bromobenzene ring was disordered over two positions with occupancy ratio 0.74: 0.26. The occupancy factors were established in earlier stages of refinement and were fixed in the final refinement cycles. The benzene ring of the smaller fraction of the bromobenzene fragment was constrained as a regular hexagon

### Crystal data

**Table:**

C_20_H_18_BrNO_4_S_2_	*F*(000) = 976
*M**_r_* = 480.38	*D*_x_ = 1.679 Mg m^−^^3^
Monoclinic, *P*2_1_/*c*	Mo *K*α radiation, λ = 0.71073 Å
Hall symbol: -P 2ybc	Cell parameters from 9904 reflections
*a* = 10.5819 (15) Å	θ = 2.3–28.1°
*b* = 13.1465 (19) Å	µ = 2.32 mm^−^^1^
*c* = 14.235 (2) Å	*T* = 100 K
β = 95.478 (2)°	Blocks, colorless
*V* = 1971.2 (5) Å^3^	0.38 × 0.33 × 0.24 mm
*Z* = 4	

### Data collection

**Table:**

Bruker KAPPA APEXII CCD diffractometer	4792 independent reflections
Radiation source: fine-focus sealed tube	4320 reflections with *I* > 2σ(*I*)
graphite	*R*_int_ = 0.031
φ and ω scans	θ_max_ = 28.3°, θ_min_ = 1.9°
Absorption correction: multi-scan (*SADABS*; Bruker, 2001)	*h* = −14→13
*T*_min_ = 0.472, *T*_max_ = 0.605	*k* = −17→17
23193 measured reflections	*l* = −18→18

### Refinement

**Table:**

Refinement on *F*^2^	Primary atom site location: structure-invariant direct methods
Least-squares matrix: full	Secondary atom site location: difference Fourier map
*R*[*F*^2^ > 2σ(*F*^2^)] = 0.038	Hydrogen site location: inferred from neighbouring sites
*wR*(*F*^2^) = 0.085	H-atom parameters constrained
*S* = 1.24	*w* = 1/[σ^2^(*F*_o_^2^) + (0.0125*P*)^2^ + 3.1006*P*] where *P* = (*F*_o_^2^ + 2*F*_c_^2^)/3
4792 reflections	(Δ/σ)_max_ = 0.001
301 parameters	Δρ_max_ = 0.52 e Å^−^^3^
0 restraints	Δρ_min_ = −0.53 e Å^−^^3^

### Special details

**Table:**

Geometry. All esds (except the esd in the dihedral angle between two l.s. planes) are estimated using the full covariance matrix. The cell esds are taken into account individually in the estimation of esds in distances, angles and torsion angles; correlations between esds in cell parameters are only used when they are defined by crystal symmetry. An approximate (isotropic) treatment of cell esds is used for estimating esds involving l.s. planes.
Refinement. Refinement of F^2^ against ALL reflections. The weighted R-factor wR and goodness of fit S are based on F^2^, conventional R-factors R are based on F, with F set to zero for negative F^2^. The threshold expression of F^2^ > 2sigma(F^2^) is used only for calculating R-factors(gt) etc. and is not relevant to the choice of reflections for refinement. R-factors based on F^2^ are statistically about twice as large as those based on F, and R- factors based on ALL data will be even larger.

### Fractional atomic coordinates and isotropic or equivalent isotropic displacement parameters (Å^2^)

**Table:**

	*x*	*y*	*z*	*U*_iso_*/*U*_eq_	Occ. (<1)
Br1	0.25875 (4)	0.31399 (3)	0.20992 (2)	0.02691 (9)	0.74
Br2	−0.06672 (10)	0.62953 (9)	0.15927 (9)	0.0346 (3)	0.26
S1	0.30298 (5)	0.58728 (4)	0.18802 (4)	0.01811 (12)	
S2	0.19193 (5)	0.55064 (5)	0.36828 (4)	0.01938 (12)	
O2	0.06261 (16)	0.55206 (14)	0.38987 (12)	0.0240 (4)	
O3	0.42299 (16)	0.56000 (13)	0.23665 (13)	0.0239 (4)	
O4	0.27382 (16)	0.63481 (13)	0.39241 (12)	0.0236 (4)	
O7	0.26821 (17)	0.55219 (13)	0.09413 (12)	0.0237 (4)	
N1	0.18608 (18)	0.54032 (15)	0.24974 (13)	0.0180 (4)	
C1	0.2899 (2)	0.72024 (17)	0.19019 (17)	0.0185 (4)	
C2	0.1987 (2)	0.76797 (19)	0.12845 (17)	0.0214 (5)	
H2	0.1401	0.7290	0.0885	0.026*	
C3	0.1949 (2)	0.87387 (19)	0.12623 (18)	0.0225 (5)	
H3	0.1324	0.9074	0.0850	0.027*	
C4	0.2817 (2)	0.93112 (18)	0.18389 (17)	0.0206 (5)	
C5	0.3712 (2)	0.88108 (18)	0.24575 (18)	0.0219 (5)	
H5	0.4299	0.9197	0.2858	0.026*	
C6	0.3756 (2)	0.77596 (19)	0.24962 (17)	0.0216 (5)	
H6	0.4363	0.7424	0.2923	0.026*	
C7	0.2787 (2)	1.04533 (19)	0.1779 (2)	0.0261 (5)	
H7A	0.3330	1.0680	0.1298	0.039*	
H7B	0.3100	1.0743	0.2392	0.039*	
H7C	0.1914	1.0681	0.1607	0.039*	
C8	0.2633 (2)	0.43825 (19)	0.41368 (17)	0.0220 (5)	
C9	0.3953 (2)	0.4307 (2)	0.42161 (17)	0.0236 (5)	
H9	0.4458	0.4872	0.4069	0.028*	
C10	0.4515 (2)	0.3396 (2)	0.45122 (18)	0.0264 (5)	
H10	0.5415	0.3342	0.4573	0.032*	
C11	0.3788 (3)	0.2558 (2)	0.47224 (19)	0.0291 (6)	
C12	0.2467 (3)	0.2666 (2)	0.4662 (2)	0.0335 (6)	
H12	0.1960	0.2107	0.4823	0.040*	
C13	0.1884 (3)	0.3570 (2)	0.4371 (2)	0.0295 (6)	
H13	0.0986	0.3634	0.4333	0.035*	
C14	0.4414 (3)	0.1552 (2)	0.4977 (2)	0.0378 (7)	
H14A	0.3871	0.1155	0.5361	0.057*	
H14B	0.5238	0.1670	0.5335	0.057*	
H14C	0.4538	0.1175	0.4399	0.057*	
C15	0.0847 (5)	0.4829 (4)	0.2018 (3)	0.0202 (11)	0.74
C16	0.1015 (5)	0.3805 (4)	0.1793 (4)	0.0237 (10)	0.74
C17	0.0016 (5)	0.3249 (3)	0.1337 (3)	0.0275 (9)	0.74
H17	0.0112	0.2545	0.1213	0.033*	0.74
C18	−0.1120 (5)	0.3739 (5)	0.1067 (3)	0.0291 (10)	0.74
H18	−0.1805	0.3365	0.0755	0.035*	0.74
C19	−0.1272 (4)	0.4763 (4)	0.1243 (3)	0.0275 (9)	0.74
H19	−0.2045	0.5095	0.1033	0.033*	0.74
C20	−0.0293 (5)	0.5305 (4)	0.1728 (3)	0.0213 (9)	0.74
H20	−0.0404	0.6005	0.1862	0.026*	0.74
C21	0.1025 (10)	0.4648 (7)	0.1985 (9)	0.012 (4)*	0.26
C22	0.1370 (9)	0.3627 (8)	0.1979 (8)	0.025 (4)	0.26
H22	0.2186	0.3422	0.2249	0.030*	0.26
C23	0.0520 (12)	0.2907 (6)	0.1578 (7)	0.038 (3)	0.26
H23	0.0756	0.2210	0.1574	0.046*	0.26
C24	−0.0673 (11)	0.3207 (8)	0.1183 (6)	0.043 (4)	0.26
H24	−0.1254	0.2715	0.0909	0.052*	0.26
C25	−0.1018 (8)	0.4227 (9)	0.1189 (7)	0.028 (3)	0.26
H25	−0.1834	0.4432	0.0919	0.034*	0.26
C26	−0.0169 (10)	0.4947 (6)	0.1590 (8)	0.024 (3)	0.26

### Atomic displacement parameters (Å^2^)

**Table:**

	*U*^11^	*U*^22^	*U*^33^	*U*^12^	*U*^13^	*U*^23^
Br1	0.03615 (19)	0.01695 (15)	0.02757 (18)	0.00531 (14)	0.00269 (14)	−0.00062 (13)
Br2	0.0257 (5)	0.0338 (6)	0.0445 (6)	0.0085 (4)	0.0044 (4)	0.0156 (5)
S1	0.0176 (3)	0.0155 (3)	0.0215 (3)	0.0006 (2)	0.0035 (2)	0.0015 (2)
S2	0.0177 (3)	0.0218 (3)	0.0185 (3)	−0.0002 (2)	0.0010 (2)	0.0017 (2)
O2	0.0198 (8)	0.0302 (9)	0.0221 (9)	0.0010 (7)	0.0028 (7)	0.0013 (7)
O3	0.0193 (8)	0.0196 (8)	0.0327 (10)	0.0025 (7)	0.0020 (7)	0.0044 (7)
O4	0.0249 (9)	0.0241 (9)	0.0215 (9)	−0.0024 (7)	0.0003 (7)	−0.0010 (7)
O7	0.0282 (9)	0.0207 (8)	0.0232 (9)	−0.0016 (7)	0.0069 (7)	−0.0007 (7)
N1	0.0181 (9)	0.0184 (9)	0.0175 (9)	−0.0026 (8)	0.0016 (7)	−0.0001 (7)
C1	0.0197 (11)	0.0141 (10)	0.0220 (11)	0.0007 (8)	0.0037 (9)	0.0023 (9)
C2	0.0208 (11)	0.0214 (12)	0.0216 (12)	−0.0004 (9)	−0.0008 (9)	0.0015 (9)
C3	0.0208 (11)	0.0221 (12)	0.0247 (12)	0.0034 (9)	0.0026 (9)	0.0065 (9)
C4	0.0199 (11)	0.0166 (11)	0.0267 (12)	0.0008 (9)	0.0094 (9)	0.0033 (9)
C5	0.0201 (11)	0.0199 (11)	0.0256 (12)	−0.0042 (9)	0.0022 (9)	0.0005 (9)
C6	0.0199 (11)	0.0213 (12)	0.0233 (12)	0.0000 (9)	−0.0001 (9)	0.0046 (9)
C7	0.0249 (12)	0.0175 (11)	0.0367 (14)	0.0030 (9)	0.0072 (11)	0.0042 (10)
C8	0.0204 (11)	0.0246 (12)	0.0207 (11)	0.0016 (9)	0.0002 (9)	0.0067 (9)
C9	0.0211 (12)	0.0281 (13)	0.0212 (12)	−0.0026 (10)	−0.0007 (9)	0.0044 (10)
C10	0.0217 (12)	0.0341 (14)	0.0231 (12)	0.0026 (10)	0.0009 (10)	0.0049 (10)
C11	0.0308 (14)	0.0323 (14)	0.0249 (13)	0.0049 (11)	0.0058 (11)	0.0100 (11)
C12	0.0281 (14)	0.0325 (15)	0.0412 (16)	0.0007 (11)	0.0094 (12)	0.0171 (12)
C13	0.0215 (12)	0.0332 (14)	0.0340 (14)	−0.0008 (11)	0.0047 (11)	0.0121 (12)
C14	0.0371 (16)	0.0335 (15)	0.0436 (17)	0.0088 (13)	0.0087 (13)	0.0165 (13)
C15	0.024 (2)	0.0188 (18)	0.019 (2)	−0.0033 (19)	0.0044 (16)	−0.0018 (15)
C16	0.029 (2)	0.023 (2)	0.020 (2)	−0.0013 (18)	0.0029 (18)	0.0049 (17)
C17	0.040 (3)	0.018 (2)	0.025 (2)	−0.011 (2)	0.006 (2)	−0.0035 (16)
C18	0.032 (3)	0.033 (3)	0.022 (2)	−0.017 (2)	0.0014 (17)	−0.004 (2)
C19	0.024 (2)	0.035 (2)	0.0238 (19)	−0.0046 (18)	0.0000 (14)	0.0022 (19)
C20	0.0201 (19)	0.026 (3)	0.018 (2)	−0.0012 (17)	0.0031 (14)	−0.0011 (18)
C22	0.050 (10)	0.012 (6)	0.013 (6)	−0.003 (6)	0.004 (6)	−0.002 (5)
C23	0.066 (10)	0.021 (6)	0.029 (7)	−0.010 (6)	0.017 (6)	0.001 (5)
C24	0.065 (13)	0.042 (10)	0.023 (7)	−0.033 (10)	0.010 (8)	0.002 (6)
C25	0.030 (7)	0.029 (9)	0.025 (6)	−0.021 (7)	−0.003 (5)	0.006 (7)
C26	0.027 (6)	0.028 (8)	0.017 (6)	0.006 (6)	0.005 (5)	0.002 (5)

### Geometric parameters (Å, °)

**Table:**

Br1—C16	1.893 (5)	C10—C11	1.393 (4)
Br2—C26	1.849 (8)	C10—H10	0.9500
S1—O7	1.4284 (18)	C11—C12	1.399 (4)
S1—O3	1.4328 (18)	C11—C14	1.509 (4)
S1—N1	1.700 (2)	C12—C13	1.384 (4)
S1—C1	1.754 (2)	C12—H12	0.9500
S2—O4	1.4271 (18)	C13—H13	0.9500
S2—O2	1.4310 (18)	C14—H14A	0.9800
S2—N1	1.688 (2)	C14—H14B	0.9800
S2—C8	1.755 (2)	C14—H14C	0.9800
N1—C15	1.431 (4)	C15—C20	1.386 (7)
N1—C21	1.475 (8)	C15—C16	1.399 (8)
C1—C6	1.389 (3)	C16—C17	1.394 (7)
C1—C2	1.391 (3)	C17—C18	1.385 (7)
C2—C3	1.393 (3)	C17—H17	0.9500
C2—H2	0.9500	C18—C19	1.382 (7)
C3—C4	1.393 (4)	C18—H18	0.9500
C3—H3	0.9500	C19—C20	1.385 (6)
C4—C5	1.395 (3)	C19—H19	0.9500
C4—C7	1.504 (3)	C20—H20	0.9500
C5—C6	1.384 (3)	C21—C22	1.3900
C5—H5	0.9500	C21—C26	1.3900
C6—H6	0.9500	C22—C23	1.3900
C7—H7A	0.9800	C22—H22	0.9500
C7—H7B	0.9800	C23—C24	1.3900
C7—H7C	0.9800	C23—H23	0.9500
C8—C13	1.390 (4)	C24—C25	1.3900
C8—C9	1.393 (3)	C24—H24	0.9500
C9—C10	1.385 (4)	C25—C26	1.3900
C9—H9	0.9500	C25—H25	0.9500
			
O7—S1—O3	120.57 (11)	C10—C11—C12	118.4 (2)
O7—S1—N1	103.36 (10)	C10—C11—C14	120.4 (2)
O3—S1—N1	108.33 (10)	C12—C11—C14	121.1 (3)
O7—S1—C1	108.94 (11)	C13—C12—C11	121.3 (3)
O3—S1—C1	107.99 (11)	C13—C12—H12	119.4
N1—S1—C1	106.86 (10)	C11—C12—H12	119.4
O4—S2—O2	120.62 (11)	C12—C13—C8	118.9 (2)
O4—S2—N1	105.45 (10)	C12—C13—H13	120.5
O2—S2—N1	105.73 (10)	C8—C13—H13	120.5
O4—S2—C8	109.51 (11)	C11—C14—H14A	109.5
O2—S2—C8	108.59 (11)	C11—C14—H14B	109.5
N1—S2—C8	105.90 (11)	H14A—C14—H14B	109.5
C15—N1—C21	12.1 (5)	C11—C14—H14C	109.5
C15—N1—S2	118.3 (2)	H14A—C14—H14C	109.5
C21—N1—S2	120.7 (6)	H14B—C14—H14C	109.5
C15—N1—S1	119.6 (2)	C20—C15—C16	119.5 (4)
C21—N1—S1	114.9 (6)	C20—C15—N1	119.7 (5)
S2—N1—S1	121.89 (12)	C16—C15—N1	120.7 (5)
C6—C1—C2	121.3 (2)	C17—C16—C15	120.2 (5)
C6—C1—S1	119.23 (18)	C17—C16—Br1	118.4 (4)
C2—C1—S1	119.32 (18)	C15—C16—Br1	121.4 (4)
C1—C2—C3	118.8 (2)	C18—C17—C16	119.0 (4)
C1—C2—H2	120.6	C18—C17—H17	120.5
C3—C2—H2	120.6	C16—C17—H17	120.5
C4—C3—C2	120.7 (2)	C19—C18—C17	121.0 (4)
C4—C3—H3	119.7	C19—C18—H18	119.5
C2—C3—H3	119.7	C17—C18—H18	119.5
C3—C4—C5	119.1 (2)	C18—C19—C20	119.8 (4)
C3—C4—C7	119.8 (2)	C18—C19—H19	120.1
C5—C4—C7	121.0 (2)	C20—C19—H19	120.1
C6—C5—C4	121.0 (2)	C19—C20—C15	120.3 (4)
C6—C5—H5	119.5	C19—C20—H20	119.9
C4—C5—H5	119.5	C15—C20—H20	119.9
C5—C6—C1	119.0 (2)	C22—C21—C26	120.0
C5—C6—H6	120.5	C22—C21—N1	120.5 (7)
C1—C6—H6	120.5	C26—C21—N1	119.2 (7)
C4—C7—H7A	109.5	C23—C22—C21	120.0
C4—C7—H7B	109.5	C23—C22—H22	120.0
H7A—C7—H7B	109.5	C21—C22—H22	120.0
C4—C7—H7C	109.5	C22—C23—C24	120.0
H7A—C7—H7C	109.5	C22—C23—H23	120.0
H7B—C7—H7C	109.5	C24—C23—H23	120.0
C13—C8—C9	121.0 (2)	C23—C24—C25	120.0
C13—C8—S2	120.03 (19)	C23—C24—H24	120.0
C9—C8—S2	118.86 (19)	C25—C24—H24	120.0
C10—C9—C8	119.0 (2)	C26—C25—C24	120.0
C10—C9—H9	120.5	C26—C25—H25	120.0
C8—C9—H9	120.5	C24—C25—H25	120.0
C9—C10—C11	121.2 (2)	C25—C26—C21	120.0
C9—C10—H10	119.4	C25—C26—Br2	118.7 (6)
C11—C10—H10	119.4	C21—C26—Br2	121.3 (6)
			
O4—S2—N1—C15	−162.2 (3)	C8—C9—C10—C11	0.6 (4)
O2—S2—N1—C15	−33.4 (4)	C9—C10—C11—C12	−2.3 (4)
C8—S2—N1—C15	81.8 (3)	C9—C10—C11—C14	175.6 (3)
O4—S2—N1—C21	−175.9 (5)	C10—C11—C12—C13	2.1 (4)
O2—S2—N1—C21	−47.0 (5)	C14—C11—C12—C13	−175.9 (3)
C8—S2—N1—C21	68.1 (5)	C11—C12—C13—C8	0.0 (4)
O4—S2—N1—S1	23.07 (16)	C9—C8—C13—C12	−1.8 (4)
O2—S2—N1—S1	151.89 (13)	S2—C8—C13—C12	175.3 (2)
C8—S2—N1—S1	−92.98 (15)	C21—N1—C15—C20	−167 (4)
O7—S1—N1—C15	−1.2 (4)	S2—N1—C15—C20	88.1 (4)
O3—S1—N1—C15	−130.2 (3)	S1—N1—C15—C20	−97.1 (4)
C1—S1—N1—C15	113.7 (3)	C21—N1—C15—C16	10 (3)
O7—S1—N1—C21	11.4 (5)	S2—N1—C15—C16	−95.3 (4)
O3—S1—N1—C21	−117.6 (5)	S1—N1—C15—C16	79.5 (4)
C1—S1—N1—C21	126.2 (5)	C20—C15—C16—C17	−4.2 (6)
O7—S1—N1—S2	173.46 (13)	N1—C15—C16—C17	179.2 (5)
O3—S1—N1—S2	44.46 (16)	C20—C15—C16—Br1	175.9 (4)
C1—S1—N1—S2	−71.67 (16)	N1—C15—C16—Br1	−0.7 (6)
O7—S1—C1—C6	−145.08 (19)	C15—C16—C17—C18	3.3 (7)
O3—S1—C1—C6	−12.5 (2)	Br1—C16—C17—C18	−176.8 (4)
N1—S1—C1—C6	103.9 (2)	C16—C17—C18—C19	−0.1 (7)
O7—S1—C1—C2	31.2 (2)	C17—C18—C19—C20	−2.3 (8)
O3—S1—C1—C2	163.77 (19)	C18—C19—C20—C15	1.4 (7)
N1—S1—C1—C2	−79.9 (2)	C16—C15—C20—C19	1.8 (6)
C6—C1—C2—C3	0.5 (4)	N1—C15—C20—C19	178.5 (4)
S1—C1—C2—C3	−175.65 (19)	C15—N1—C21—C22	−159 (4)
C1—C2—C3—C4	0.8 (4)	S2—N1—C21—C22	−77.5 (7)
C2—C3—C4—C5	−1.4 (4)	S1—N1—C21—C22	84.8 (7)
C2—C3—C4—C7	177.8 (2)	C15—N1—C21—C26	14 (3)
C3—C4—C5—C6	0.8 (4)	S2—N1—C21—C26	95.9 (7)
C7—C4—C5—C6	−178.5 (2)	S1—N1—C21—C26	−101.7 (6)
C4—C5—C6—C1	0.5 (4)	C26—C21—C22—C23	0.0
C2—C1—C6—C5	−1.2 (4)	N1—C21—C22—C23	173.4 (11)
S1—C1—C6—C5	174.99 (19)	C21—C22—C23—C24	0.0
O4—S2—C8—C13	151.7 (2)	C22—C23—C24—C25	0.0
O2—S2—C8—C13	18.1 (3)	C23—C24—C25—C26	0.0
N1—S2—C8—C13	−95.1 (2)	C24—C25—C26—C21	0.0
O4—S2—C8—C9	−31.2 (2)	C24—C25—C26—Br2	−179.5 (8)
O2—S2—C8—C9	−164.8 (2)	C22—C21—C26—C25	0.0
N1—S2—C8—C9	82.1 (2)	N1—C21—C26—C25	−173.5 (11)
C13—C8—C9—C10	1.6 (4)	C22—C21—C26—Br2	179.5 (8)
S2—C8—C9—C10	−175.6 (2)	N1—C21—C26—Br2	6.0 (8)

### Hydrogen-bond geometry (Å, °)

**Table:**

*D*—H···*A*	*D*—H	H···*A*	*D*···*A*	*D*—H···*A*
C5—H5···O3^i^	0.95	2.45	3.199 (3)	135.

Symmetry codes: (i) −*x*+1, *y*+1/2, −*z*+1/2.
